# Field Demonstration of Real-Time Wind Turbine Foundation Strain Monitoring

**DOI:** 10.3390/s18010097

**Published:** 2017-12-31

**Authors:** Tim Rubert, Marcus Perry, Grzegorz Fusiek, Jack McAlorum, Pawel Niewczas, Amanda Brotherston, David McCallum

**Affiliations:** 1 Doctoral Training Centre in Wind and Marine Energy Systems, University of Strathclyde, Glasgow G1 1XQ, UK; 2 Department of Civil & Environmental Engineering, University of Strathclyde, Glasgow G1 1XJ, UK; m.perry@strath.ac.uk; 3 Department of Electronic & Electrical Engineering, University of Strathclyde, Glasgow G1 1XQ, UK; g.fusiek@strath.ac.uk (G.F.); g.fusiek@strath.ac.uk (J.M.); p.niewczas@strath.ac.uk (P.N.); 4 Scottish and Southern Energy (SSE), Glasgow G2 6AY, UK; amanda.brotherston@sse.com (A.B.); david.mccallum@sse.com (D.M.)

**Keywords:** wind turbine foundation, structural health monitoring, finite element model, reinforcement strain, fibre Bragg grating

## Abstract

Onshore wind turbine foundations are generally over-engineered as their internal stress states are challenging to directly monitor during operation. While there are industry drivers to shift towards more economical foundation designs, making this transition safely will require new monitoring techniques, so that the uncertainties around structural health can be reduced. This paper presents the initial results of a real-time strain monitoring campaign for an operating wind turbine foundation. Selected reinforcement bars were instrumented with metal packaged optical fibre strain sensors prior to concrete casting. In this paper, we outline the sensors’ design, characterisation and installation, and present 67 days of operational data. During this time, measured foundation strains did not exceed 95 μϵ, and showed a strong correlation with both measured tower displacements and the results of a foundation finite element model. The work demonstrates that real-time foundation monitoring is not only achievable, but that it has the potential to help operators and policymakers quantify the conservatism of their existing design codes.

## 1. Introduction

Structural health monitoring of wind turbine components is observed as a potential area to improve operations and maintenance procedures, monitor fatigue damage, and thus assist lifetime extension decision making [1,2,3,4]. Monitoring requires sensor data, and the transformation of fibre Bragg gratings (FBGs) into sensing transducers is an established, yet active field of research. Review papers discussing the fundamental theory and early industrial deployments of FBGs include those by Rao [5], Kersey [6], as well as Hill and Meltz [7], who highlight applications within structural health monitoring. Higuera et al. [8] present optical fibre sensors for modern structural health monitoring applications, emphasising examples of best practice for asset-monitoring in industries ranging from energy to transportation. Indeed, both Higuera et al. and Ciang et al. [9] highlight the value of FBG sensors for fatigue and damage-detection in wind turbine components. Previous research in this area has included FBG strain gauges in blades [10], wind turbine towers [11], foundation piles [12], gearboxes [13,14], accelerometers [15], and for temperature and generator current measurements [16,17,18]. Higuera specifically notes that the instrumentation of onshore, concrete wind turbine foundations with optical fibre sensors could provide valuable structural health monitoring data during construction and operation [8].

Some work has already been done in this area. Currie et al. [19,20] evaluated damage at the can-foundation interface by monitoring the tower’s vertical displacement, while Bai et al. [21] evaluated foundation crack development by means of embedding sensors in pre-cured concrete blocks. In Bai’s project, the foundation was further equipped with hollow steel tubes that are vertically inserted, allowing horizontal ultrasonic testing aimed at identification of the location of crack initiation. Perry et al. [22] and McAlorum et al. [23], meanwhile, have recently retrofitted FBG monitoring systems to existing, cracked wind turbine foundations, while Fujiyama et al. [24] have analysed the stress state of tower-foundation connections. At the time of writing, strain monitoring systems have not yet been installed *within* a wind turbine foundation, so there are few data to verify the stress distributions within the foundation’s reinforcement cage during operation.

In this paper, we outline a field instrumentation and FBG monitoring campaign for the reinforcement cage of a wind turbine foundation and tower. Optical strain gauges were installed along the turbine’s predominant wind direction and results were compared against a simplified finite element model. This field trial has demonstrated that the real-time monitoring of internal foundation strains is achievable. With refinement, such a system could allow operators to directly measure foundation rebar stress distributions during wind loading. This may allow operators and policymakers to verify the conservatism of existing foundation models and design codes, in turn reducing the financial and environmental costs associated with overdesign and overengineering.

The remainder of this paper is structured as follows. Section 2 presents the methodology including the finite element analysis (FEA) of the foundation before outlining the experimental process inclusive of: (i) sensor design and packaging; (ii) sensor positioning within the foundation, and (iii) the installation procedure of the field trial. Section 3 presents the sensor measurement results and comparison to the FEA. Findings are subsequently validated in Section 4. A discussion is provided in Section 5 along with opportunities for future work, before concluding remarks in Section 6.

## 2. Methodology

The foundation instrumented in this work is a submerged gravity foundation consisting of a reinforced concrete slab with a circular pedestal upstand. The foundation is symmetric about any axis in plan and the main slab has a haunched profile in section with the depth increasing at a constant rate towards the foundation centre. Due to confidentiality requirements, the foundation geometry and turbine type is not disclosed.

This Section presents (i) sensing options and FBG operating principles; (ii) the foundation FE model; (iii) sensor design and packaging; (iv) sensor positioning within the foundation, and (v) the field installation procedure.

### 2.1. Sensor Application

#### 2.1.1. Implementation Options

The objective of this work is to monitor strain levels in the reinforcement cage of a concrete slab foundation of an onshore wind turbine. The sensor network in question should fit the following requirements:

- Long lifetime under dynamic loading
- Multiple strain sensors throughout the foundation
- Resistant to harsh environmental factors (temperature, alkalinity, vibration)
- Unconstrained and rapid installation
- Geometrically small to minimise disturbance of stress transfer between concrete and reinforcement
- Adequate sampling frequency to encompass the turbine’s dynamic range (>50 Hz)

Overall, optical fibre sensors have become a common sensing technology in structural health monitoring systems due to their ability to meet the requirements of many applications. Key advantage are the technology’s unique electro-magnetic interference immunity, that is a concern when instrumenting power generating structures and the ability for multiplexing [5,25]. Optical sensors range from affordable, high resolution point sensors (e.g., fibre Fabry-Perot) to more expensive, fully distributed schemes (e.g., Brillouin fibre sensing) [26]. In this work, we opted for a series of wavelength-division multiplexed FBG sensor lines. These were able to provide quasi-distributed strain and temperature sensing across the foundation with a suitable trade-off between cost and sensor performance. Furthermore, as each FBG is a point sensor, the system can be interrogated rapidly and without ambiguity about sensor location.

#### 2.1.2. Fibre Bragg Gratings

FBGs are an established point strain sensing instrument. An optical fibre is side-illuminated with diffracted ultra-violet light to induce a periodic variation in refractive index, known as a grating. Broadband light incident on this grating causes a short-band reflection around a particular wavelength Bragg peak, $\lambda_{B}$, dependent on the period between gratings, Λ, and effective index, $\eta_{e}$:

(1) $$\lambda_{B}=2\eta_{e}\Lambda$$

This wavelength peak is surveyed using an interrogation system that allows frequent measurements of any variation, $\Delta \lambda_{B}$. Standalone FBG wavelength peak changes are governed by temperature fluctuations, ΔT; however, bonding said FBG to a structure allows simultaneous measurement of change of structural strain, Δϵ:

(2) $$\frac{\Delta \lambda_{B}}{\lambda_{B}}=K_{\epsilon }\Delta \epsilon +K_{T}\Delta T$$

where $K_{\epsilon }$ and $K_{T}$ are the strain and temperature coefficient respectively. Therefore, it is common for a bonded FBG to be accompanied by an isolated FBG to allow for temperature compensation.

### 2.2. Finite Element Model

**Figure 1:**
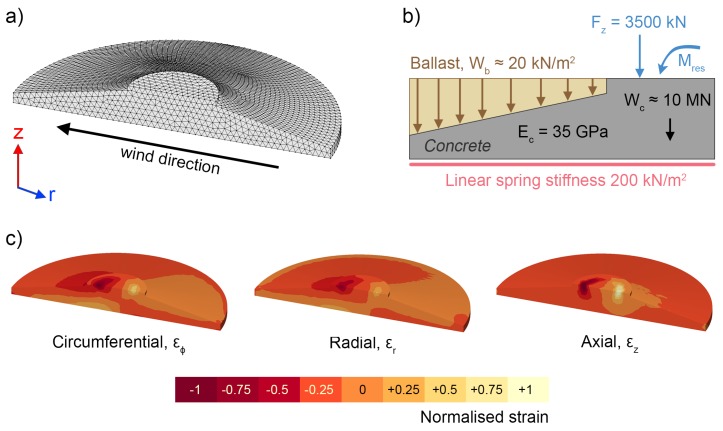
FE model (**a**) mesh and wind vector; (**b**) boundary conditions; and (**c**) typical normalised strain profiles in circumferential, radial and axial directions.

The foundation FE model’s mesh and boundary conditions are illustrated in Figure 1a,b respectively. The cylindrical coordinate system used is shown in Figure 1a. Only one half of the model is shown for simplicity, as geometry and loading conditions are symmetrical. The model, which was constructed using LUSAS, has an irregular mesh made up of linear tetrahedral stress elements (size 0.4 m). It includes several dead loads: the weight of the concrete foundation, $W_{c}$, and tower, $F_{z}$, along with the weight of the ballast, $W_{b}$. Soil-structure interactions were modeled using a linear spring constant on the bottom surface of the base, with lift-off occurring for positive vertical displacements. The concrete was assumed to have a constant elastic modulus of $E_{c}$ = 35 GPa, a Poisson’s ratio of $\nu_{c}=0.2$ and a mass density of $\rho_{c}=2500$ kg/m^3^. Horizontal slippage of the foundation, bolt loads and hydrostatic uplift were not included in the model, nor were torsional moments about the tower axis.

Strains within the foundation were studied for overturning moments, $M_{res}$, ranging from 0 MNm to 30 MNm. As the foundation’s reinforcement was not included in the model, absolute stress and strain values are not accurate. However, comparisons of fractional strain changes between locations or between load cases are valid, and these were used to inform sensor placement. Typical circumferential, radial and axial strain profiles are shown in Figure 1c, where negative numbers represent compression. As expected, on the side of the foundation opposite to the prevailing wind direction, there are compressive stresses on the top surface, while the bottom surface is under tension.

### 2.3. Field Trial Preparation

#### 2.3.1. Sensor Design and Packaging

**Figure 2:**
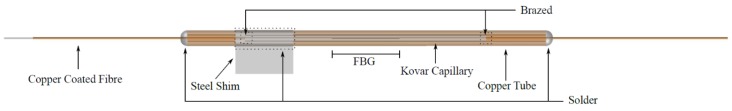
Brazed kovar capillary and soldered copper tube with optional shim temperature sensor.

**Figure 3:**
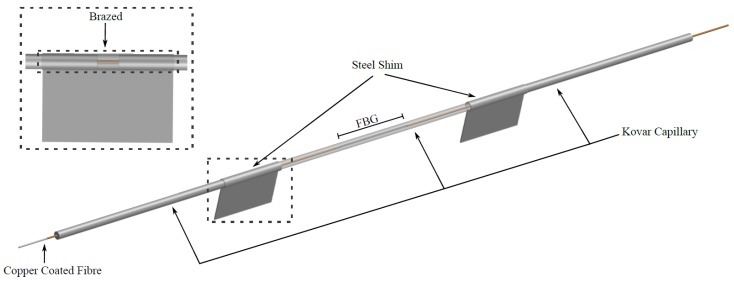
FBG strain gauge.

The FBG based temperature and strain gauge designs adopted in this project are illustrated in Figure 2 for the temperature sensor and in Figure 3 for the strain gauge. Their design and characteristics suited the environmental conditions best compared to other variants as analysed in [27,28,29].

The temperature sensor consists of a 7 mm-long FBG inscribed in copper coated fibre, placed within a 25 mm-long kovar capillary (ID 200 μm; OD 700 μm). The capillary is hermetically sealed by brazing during an induction heating process [30,31]. To isolate temperature measurements from any potential external mechanical stresses, the kovar capillary is positioned in a 40 mm long copper tube (ID 1620 μm; OD 2370 μm) and then sealed at both ends with solder as schematically illustrated in Figure 2. For the temperature sensors, the steel shim highlighted in Figure 2 was not included so that the temperature sensor could be housed within the cable used to address the sensors.

**Figure 4:**
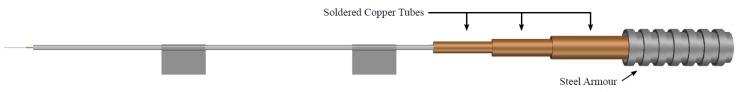
Optical strain gauge’s designed end.

The strain sensors have a similar configuration, but two 10 mm kovar capillaries are added at both ends where the joints are located in the centre of the steel shims, as illustrated in Figure 4. Each of these joints, comprised of both kovar capillaries and the steel shim, are then brazed in single stages. The use of the steel shim enabled spot welding of the sensor onto an identified location of the foundation reinforcement cage. The optical strain gauge thus has a total length of 47 mm, and a maximum width at the location of the steel shim of approximately 8.5 mm, and a height of 0.8 mm with both steel shims located 15 mm apart.

Due to the step-wise installation of the reinforcement cage, it was not practical to prepare complete interconnected sections in advance. Therefore, sensors were manufactured as modules, either as a single optical strain gauge or a strain gauge coupled with a temperature sensor. This modular configuration allowed sensors to be installed in a flexible manner as the construction of the reinforcement cage progressed. Each module was connected and sealed against water ingress during the field installation. Note that to increase redundancy in this experimental installation, each module was connected at both ends. This enabled interrogation from either end and thus increased the likelihood of survival of the sensors should some addressing cables be damaged. Modules were terminated with Diamond DiaLink connectors. As these connectors have a small diameter, placement within steel armour (ID: 6 mm) was possible as an effective means of protection. Between modules, tactical tight buffered optical cable was used, as this is suitable for deployment in the harsh environment that concrete pouring and curing presents. The length of the optical strain gauge was required to be as small as possible to fit in most locations, particularly between orthogonally positioned steel reinforcement, meaning the geometry of the sensor placement onto the reinforcement required a step wise increase in diameter to prevent the kovar capillary from bending. Such an approach also allowed a robust connection of each module’s end with the steel armour that was crimped at each side and sealed with shrink sleeve to prevent water ingress. The actual design is illustrated in Figure 4 where three copper tubes with a gradually increasing diameter, each with a length of 20 mm, were soldered in place and the steel armour was crimped to the highest-diameter copper tube.

**Figure 5:**
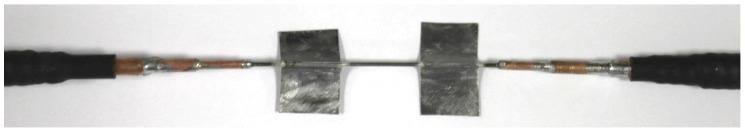
Manufactured FBG strain gauge.

Figure 5 illustrates the bespoke in-house manufactured FBG strain gauge. Despite applying flux to prevent oxidisation of the steel shim during the brazing process, usually some degree of debris remained. As this could reduce the quality of spot-welding the sensor to the steel reinforcement, the steel shim was cleaned with fine sandpaper (P400) [27].

The temperature sensors were spliced on one end of the optical strain gauge and positioned within the steel armour as illustrated in Figure 2. Within the vicinity of the temperature sensor, it was important to bind the cable to the rebars without any bends, as this could change the characteristics of the sensor.

In addition to the foundation sensor deployment, the bottom wind turbine tower section was equipped with FBG based strain gauges to monitor the fore-aft and side to side tower movement. The aim was to monitor and relate tower movement data to foundation strain measurements, via a temporally synchronised acquisition system. Therefore, in total 4 optical strain gauges, two along the prevailing wind direction (one at each side, hence 180 rotated) and two 90 degrees rotated are attached to a bare tower section patch with a cement based fast curing epoxy (Kyowa CC-33A).

#### 2.3.2. Thermal and Mechanical Characterisation

All sensors were placed in a Thermotron S-16 environmental chamber and cyclically heated in 10 ${}^{\circ }$C increments between 25 ${}^{\circ }$C and 65 ${}^{\circ }$C to characterise their temperature response. The chamber was programmed to stabilise for one hour at each temperature step. To improve accuracy of the reference temperature reading, the environmental chamber was equipped with a Pico SE012 temperature probe with an accuracy of ±0.03 ${}^{\circ }$C.

**Figure A1:**
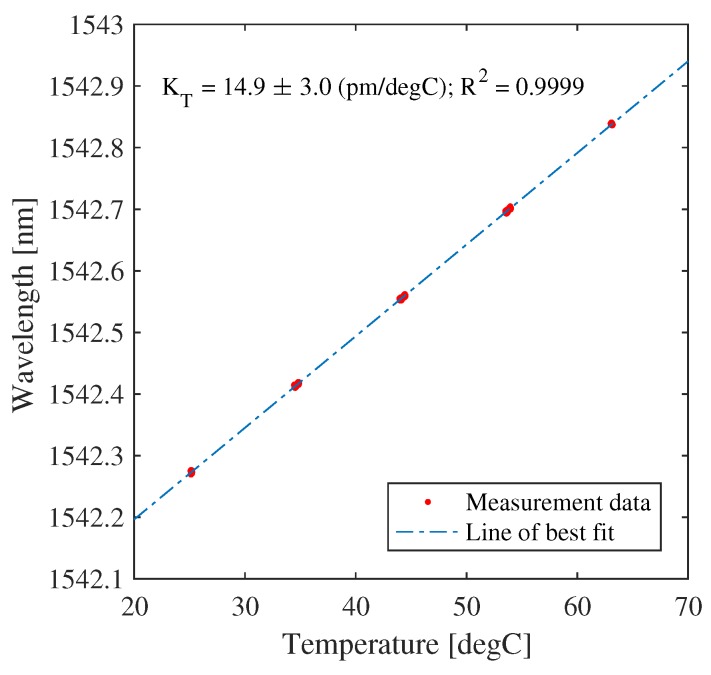
Temperature characterisation of foundation temperature sensor.

**Figure A2:**
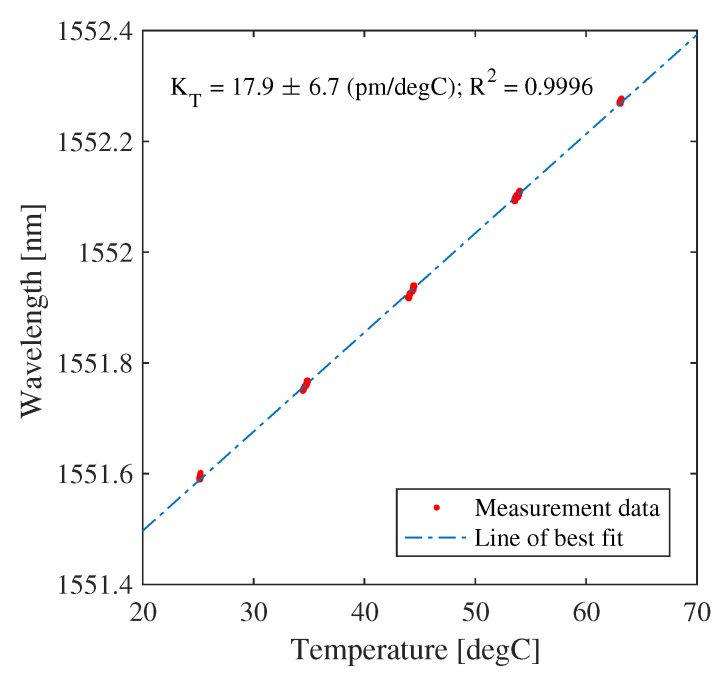
Temperature characterisation of foundation FBG strain gauge.

**Figure A3:**
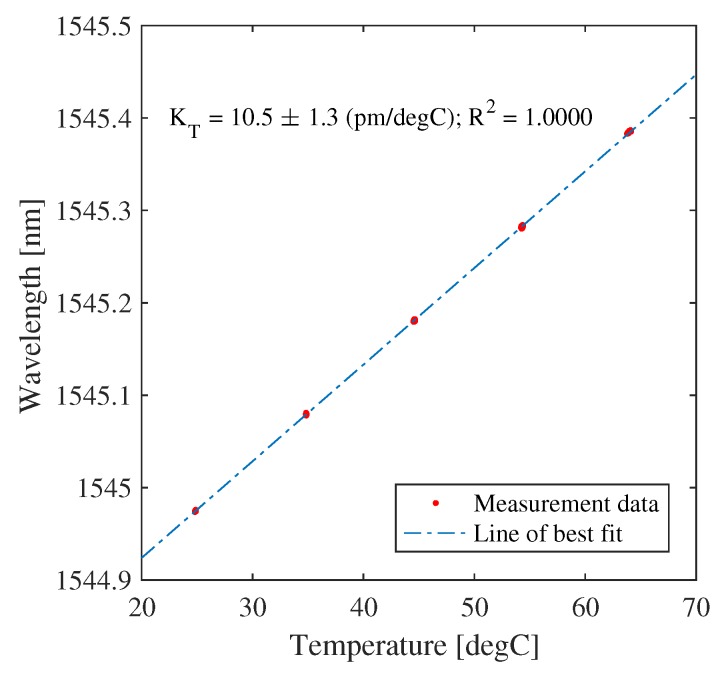
Temperature characterisation of tower temperature sensor.

The metal packaged foundation temperature sensors’ sensitivity ranged from 14.4 to 16 pm/${}^{\circ }$C (characterisation illustrated in Figure A1 of the Appendix A), the metal packaged optical strain gauges’ sensitivity for the foundation ranged from 17.2 to 18.6 pm/${}^{\circ }$C (Figure A2 of the Appendix A), while the tower temperature sensors’ sensitivity is 10.5 pm/${}^{\circ }$C (FBG attached only on one side; Figure A3 of the Appendix A).

While the temperature sensors remained in their characterised state, the FBG strain gauges required further testing to evaluate the impact that being mounted to a steel substrate has on thermal expansion. The latter is in the case of a B500B reinforcement bar for the foundation application and S355 steel for the tower application, respectively. Therefore, a different set of equally manufactured foundation and tower strain sensors were attached to their corresponding material samples, with an identical installation process compared to the field deployment. Once attached, the mounted optical strain gauges were temperature characterised in the above-mentioned fashion.

**Figure A4:**
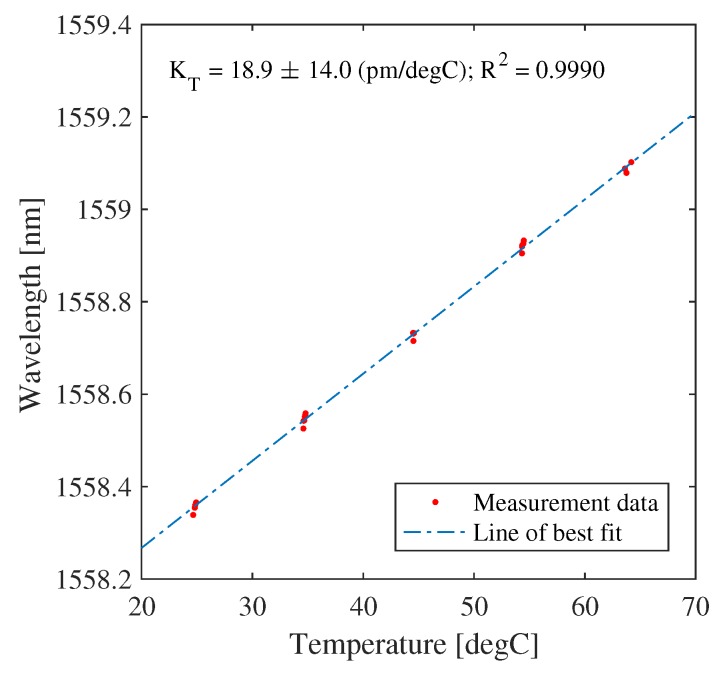
Temperature characterisation of metal packaged optical strain gauge attached to a metal sample (1/2).

**Figure A5:**
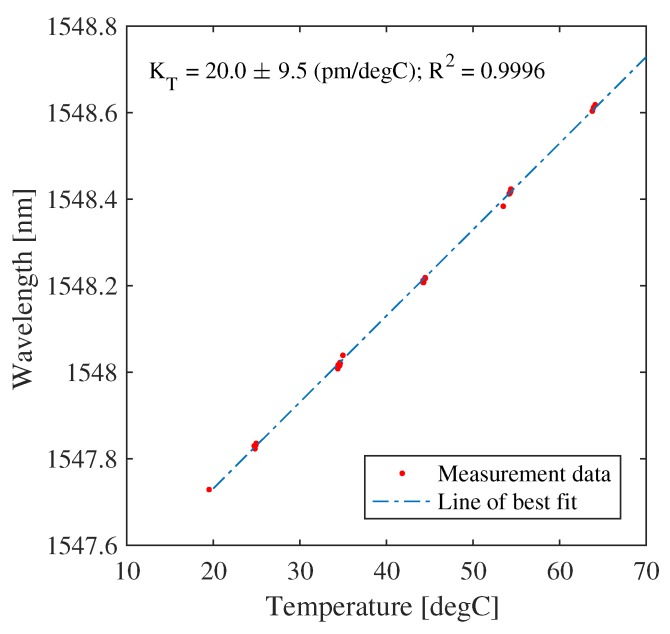
Temperature characterisation of metal packaged optical strain gauge attached to a metal sample (2/2).

**Figure A6:**
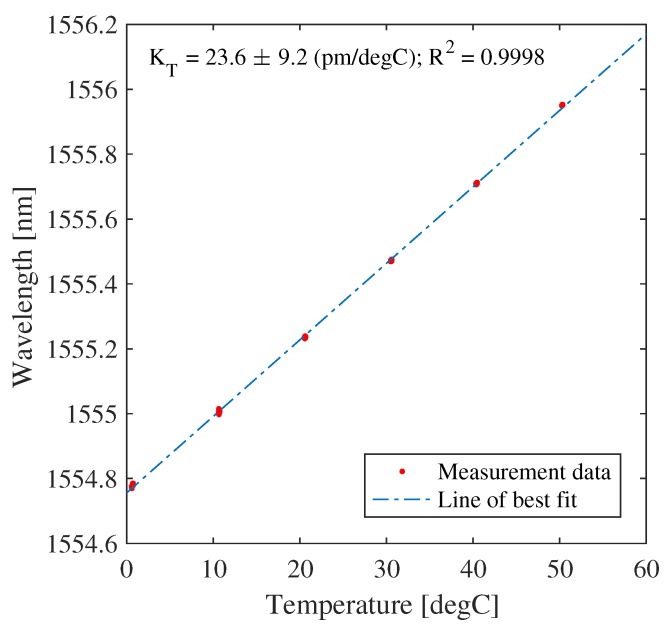
Temperature characterisation of tower FBG strain gauge attached to a metal sample.

The foundation FBG strain gauge attached to a B500B reinforcement bar resulted in an increased thermal sensitivity of 18.9 to 20 pm/${}^{\circ }$C (steel has greater thermal expansion than kovar; Figure A4 and Figure A5 of the Appendix A), while the tower FBG strain gauge epoxied to a S355 steel sample resulted in a thermal sensitivity of 23.6 pm/${}^{\circ }$C (Figure A6 of theAppendix A). Concerning the prior, since concrete has a comparable expansion coefficient to steel, the sensor-reinforcement temperature characteristics are applied for the foundation’s temperature compensation. As identified by Zhu and Ertekin [32] thermal properties of disordered materials may exhibit a dependency on strain and sample size at nanoscales; however the applied sensor designs are first governed by their metal packaging and second designed for deployment at greater-scales, hence suitable for linear characterisation [33].

Before the field deployment and after thermal characterisation, the mounted foundation and tower FBG strain gauges were subsequently subjected to static laboratory testing to identify the strain sensitivity as exemplified in [27,28]. Knowledge of the sensor sensitivity allows relation of observable changes in the centre wavelength of the field measurement campaign to strain, and therefore stress, of the instrumented component. The characterized foundation FBG strain gauge produced an average sensitivity of 0.80 nm/mϵ whereas the tower FBG strain gauge showed an increased sensitivity of 0.89 nm/mϵ (Table A1).

Overall, results from the thermal and mechanical characterisation appear sensible; i.e., it is expected that the epoxied FBG which is directly in contact with the component surface has the highest transfer rates, which is confirmed by the thermal as well as mechanical characterisation. In addition, the foundation metal packaged optical strain gauge has a higher thermal sensitivity than the metal packaged temperature sensor as well as compared to a non-attached state due to the greater thermal expansion of the steel reinforcement.

### 2.4. Assessment of Sensor Placement and Orientation

**Figure A7:**
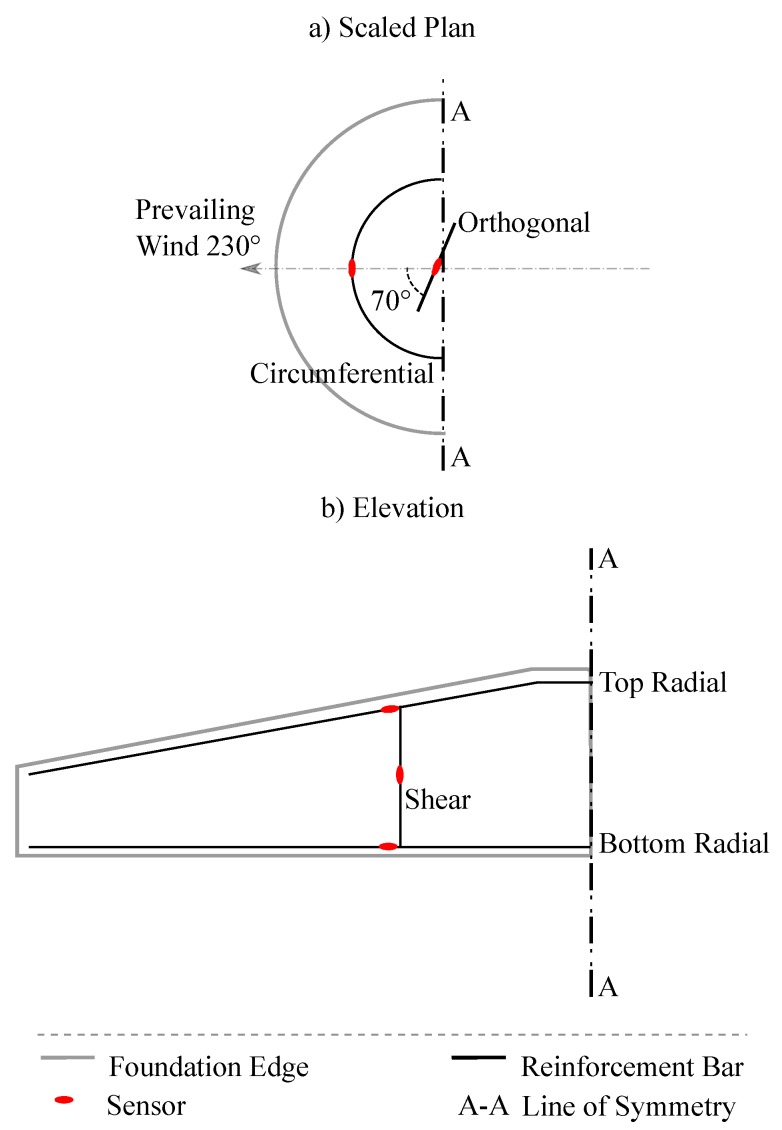
Schematic overview of sensor locations. The A-A line is the foundation centre. Red dots indicate the strain gauge placement within the foundation.

In total, the project aimed to install ten foundation FBG strain gauges (five at each side of the prevailing wind direction), six foundation temperature sensors, four tower FBG strain gauges, and four tower temperature sensors. The location identification assessment of the FBG strain gauge placement within the reinforcement cage was based on the following information: (i) the FE foundation model (Section 2.2); (ii) consultation with foundation designers; (iii) foundation geometry and installation accessibility (CAD Drawings); (iv) installation sequence of the reinforcement cage, and (v) the wind rose & site assessment. Figure A7 in the Appendix A illustrates a schematic overview of the proposed sensor placement. The five red dots indicate the sensor location at each side along the prevailing wind direction. The grey lines indicate the foundation edges, whereas the black lines indicate reinforcement bars. The top drawing of Figure A7 displays a scaled plan, enabling view of the orthogonal and circumferential reinforcement bars. The bottom drawing of Figure A7 displays the elevation view of the foundation, enabling visualization of sensor positions on both top and bottom radial bars, as well as the shear link in between.

### 2.5. Field Installation

**Figure 6:**
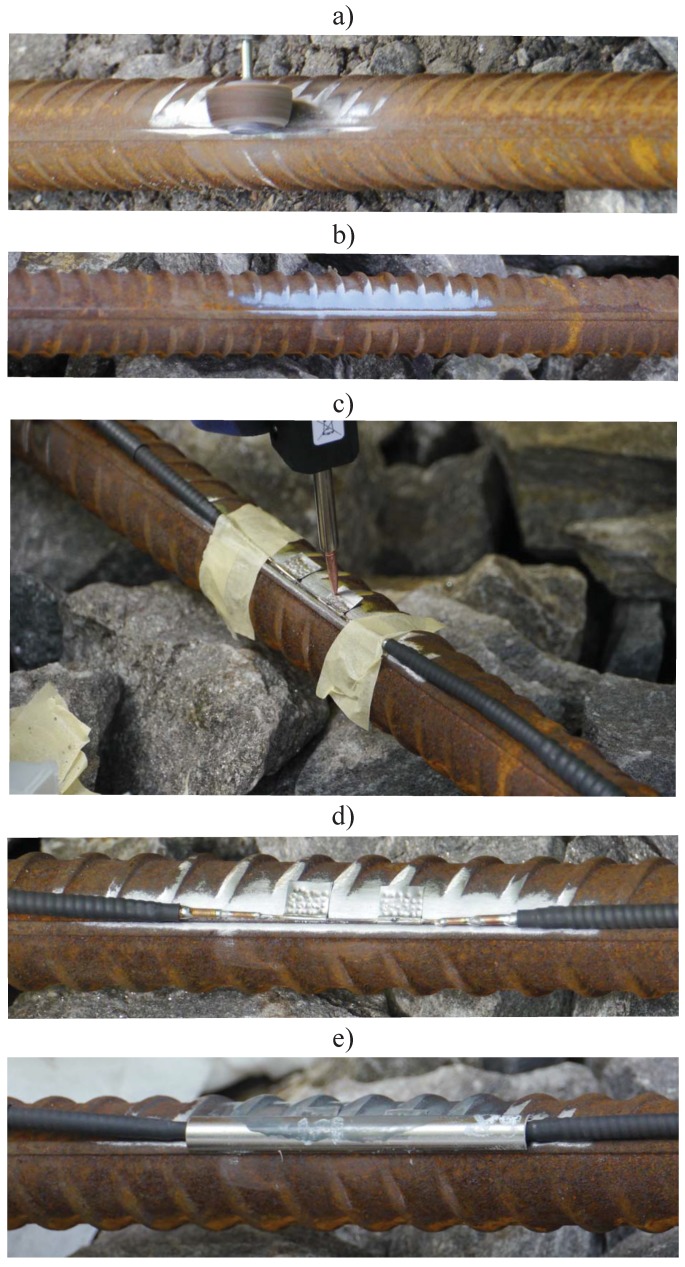
Sensor installation overview—(**a**) surface preparation; (**b**) prepared surface; (**c**) sensor attachment (spot welding); (**d**) attached sensor; (**e**) sensor protection.

As an outcome of the sensor placement assessment of Section 2.4, the intended sensor location was known. On site, the sensor area was marked with spray paint after measurement to ease installation. Surface preparation is critical to ensure reliable sensor attachment using spot welding [27]. Therefore, care was taken to prepare the surface with drill sanding bits, initially with P80 grit and subsequently with P400. The overall installation process in graphically illustrated in Figure 6.

Figure 6a illustrates the surface preparation process. A fully prepared reinforcement bar is shown in photograph (b). Once the surface was prepared, the sensor was attached with a portable spot welding unit as shown in Figure 6c. Photograph (d) illustrates the finished result—the sensor attached onto the reinforcement bar. To prevent any potential damage to the sensor, a stainless-steel half tube was positioned on top of the strain gauge as well as copper tubes. Along the half tube, silicone was applied to avoid distorting the instrument’s characteristics. The half steel tube is designed to protect the sensor from any excessive impacts or forces, for example, due to workers stepping on the transducers or if the concrete vibrating poker makes direct contact with the strain sensor during the concrete pour. The half steel tube placement is further illustrated in photograph (e) of Figure 6.

After the concrete pour, the multiplexed strain and temperature sensors were checked; however, it became evident that only the circumferential and shear link survived the installation, including their corresponding temperature sensors (due to the remote turbine location, the sensors’ interrogation during the pour and subsequent curing was not viable).

**Figure A8:**
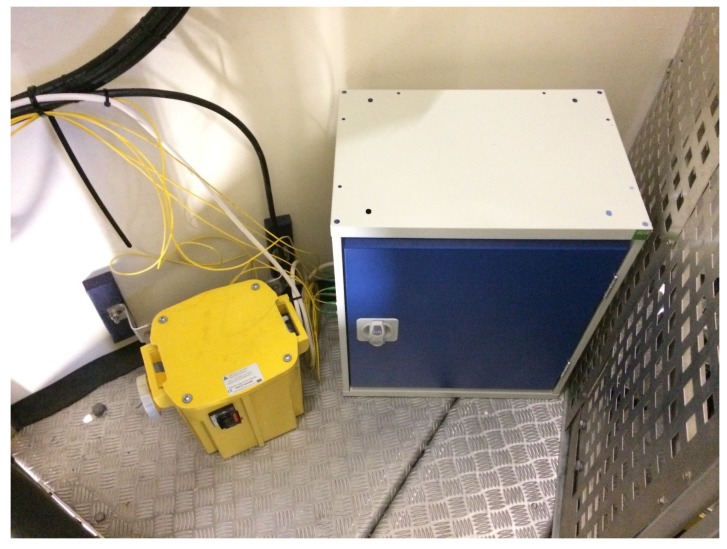
Installed interrogation system.

During the measurement campaign, the sensors were interrogated with a Smart Fibres-SmartScan unit at a sampling frequency of 100 Hz. This relatively high frequency was selected to initially monitor the higher frequency spectrum, although in terms of fatigue and cumulative damage assessment, the lower frequency spectrum is of higher importance. Generated data was saved on a locally installed computer and periodically transferred to a hard drive. The system is further equipped with an uninterruptible power supply (UPS) unit to bridge short-term power outages (up to 6 h). The installed interrogation system is further illustrated in Figure A8 of the Appendix A.

## 3. Results

Due to access restrictions, the team could not frequently access the turbine to monitor the foundation sensors during curing and foundation installation. As such, only two dates were available to evaluate the impact of the turbine weight and rheological strain (creep and shrinkage) [34]. The first date was shortly after concrete curing, while the second date was shortly before turbine commissioning. Results show that both foundation strain sensors experience a tension of around 360 μϵ that is in the same order of magnitude as identified by Bai et al. [21]. It is worth noting that, as expected, the foundation temperature sensors did not experience such a transition.

The following Section presents data readings from 67 days of measurement recorded between June and August 2017. Before, the FBG strain gauge data was analysed, their signal was temperature compensated with the pre-characterised temperature sensors as presented in Section 2.3.2 and outliers were filtered out, the latter likely to be introduced by FBG peak detection errors in the interrogation system. In order to translate the change in recorded wavelength to a strain and hence stress value, the field trial’s set-up was replicated in a laboratory to identify the sensor’s operational sensitivity for the tower and foundation sensors as highlighted in Section 2.3.2.

### 3.1. Sensor Data

**Figure 7:**
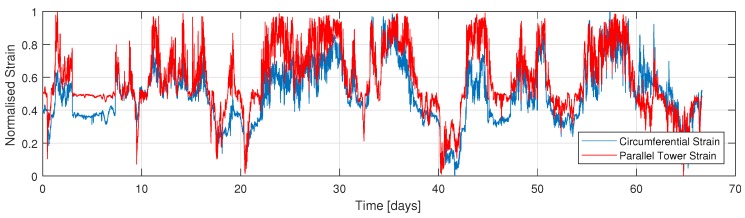
Time series of normalised strain of circumferential foundation and parallel tower sensor. Each sensor is normalised separately.

**Figure 8:**
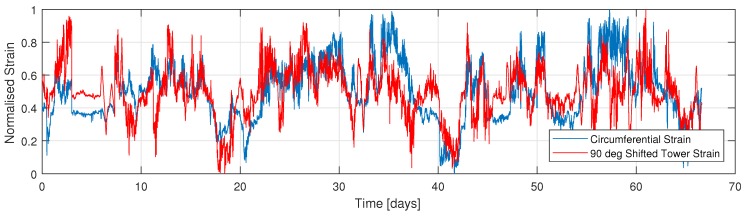
Time series of normalised strain of circumferential foundation and 90 deg rotated tower sensor. Each sensor is normalised separately.

Figure 7 presents the time series of the normalised strain of the circumferential strain sensor in comparison to the parallel tower strain sensor (T1). In addition, Figure 8 presents the circumferential’s time series, although in comparison to the 90 degree rotated tower strain data (T2).

**Figure 9:**
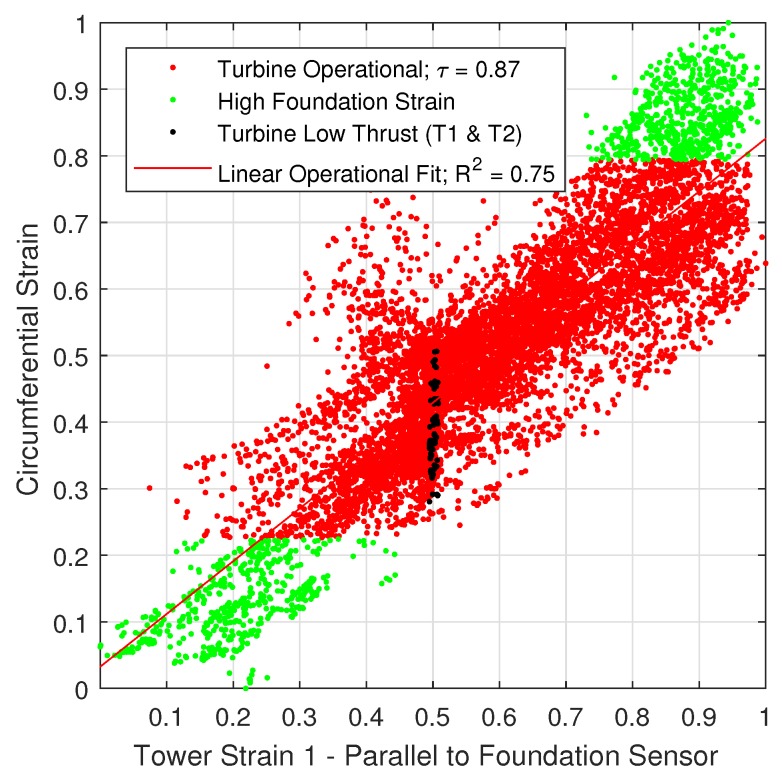
Normalised correlation of shear link strain with parallel tower sensor.

**Figure 10:**
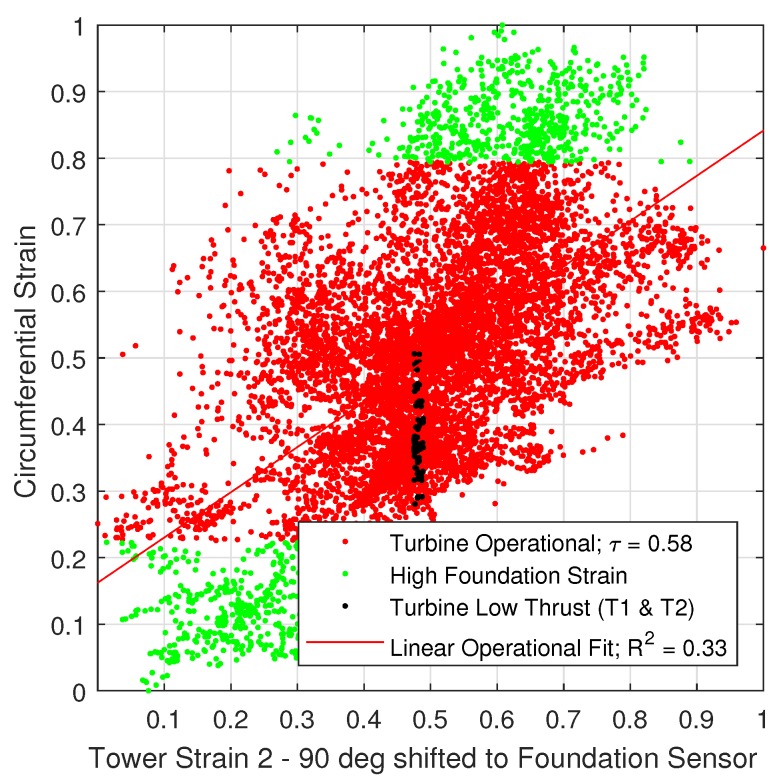
Normalised correlation of shear link strain with 90 deg rotated tower sensor.

The operational data was further tested under the hypothesis of an underlying normal distribution by means of the Kolmogorov-Smirnov and Lilliefors test. Both tests confirmed that the strain data is normally distributed. Therefore, the Pearson correlation coefficient, τ was selected to determine the degree of correlation of the operational data [35]. The circumferential sensor correlation is illustrated in Figure 9 and Figure 10. In addition, the operational data was fitted to a linear function and the fit was tested by means of $R^{2}$. Findings are further classified in three operational modes of the turbine: (i) operational (red data points); (ii) under vertical dead load tower strains (T1 and T2), and (iii) under high foundation strain (green data points). Each point represents the 10-min mean of 1599 h of measurements.

**Figure A9:**
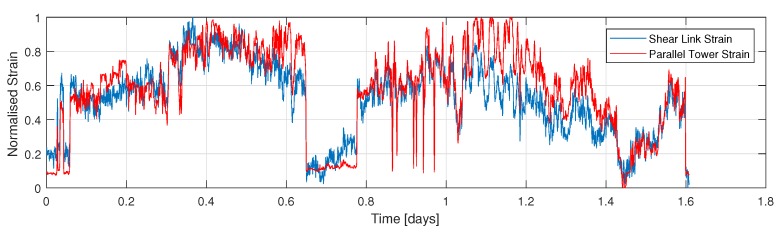
Time series of normalised strain of shear foundation and parallel tower sensor. Each sensor is normalised separately.

**Figure A10:**
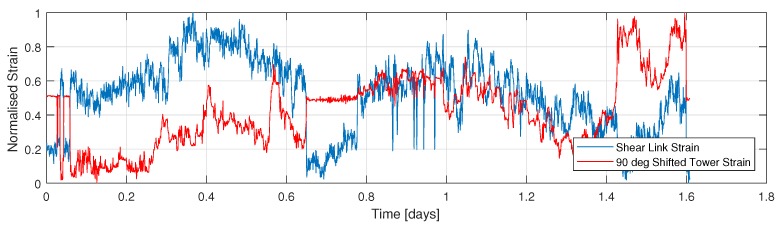
Time series of normalised strain of shear foundation and 90 deg rotated tower sensor. Each sensor is normalised separately.

**Figure A11:**
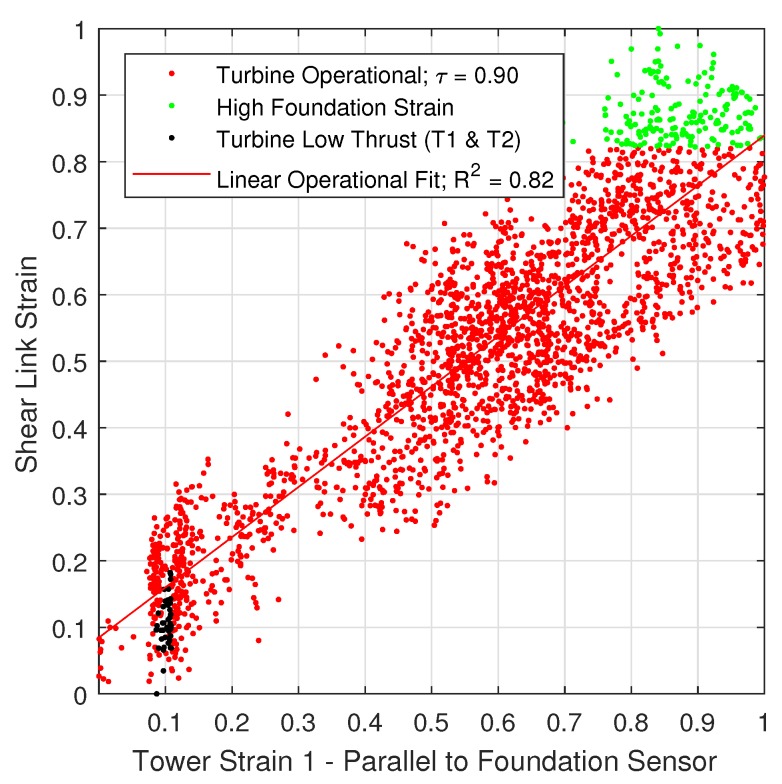
Normalised correlation of shear link strain with parallel tower sensor.

Results show a strong positive correlation between the measured parallel tower strain with a correlation coefficient, τ of 0.87, whereas a moderate positive correlation with the 90 degrees rotated tower strain (τ=0.58). With respect to the shear link strain measurements, unfortunately three days after turbine commissioning a peak detection error occurred. Therefore, 1.6 days of operational data is available. Overall, findings are slightly different for the shear link strain measurements as illustrated in Figure A9, Figure A10 and Figure A11 of the Appendix A. Findings also show a strong positive correlation (τ=0.90) with the parallel tower FBG strain gauge and a weak positive correlation (τ=0.5) with the 90 degrees rotated tower sensor, respectively.

**Figure 11:**
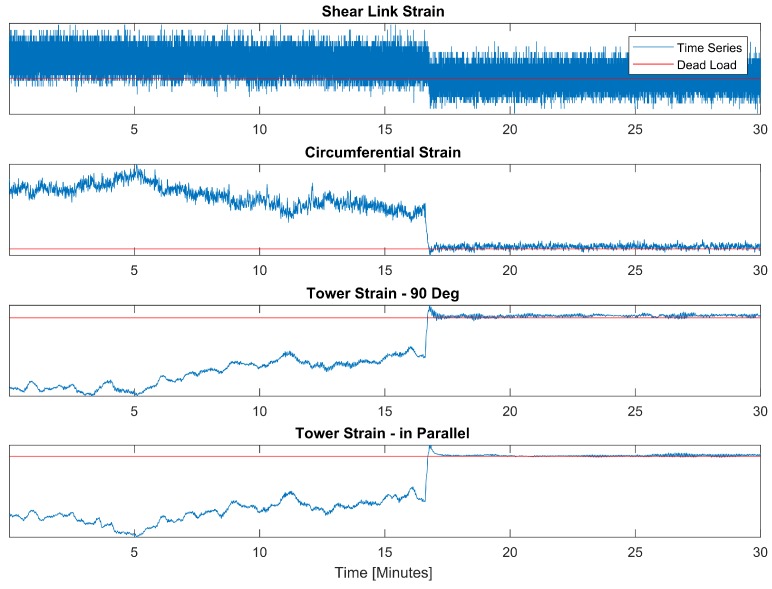
Time series of sensor measurements. The vertical axis represents the change in wavelength and the red line indicates dead load condition. Due to confidentiality the *y* axis is not disclosed.

Figure 11 further displays a noise component that is picked up in the foundation strain signals. This noise first appeared when the turbine got energised and is further picked up in the foundation temperature sensors. The signal component periodically varies in frequency and magnitude and does not coincide with any of the turbine’s natural modes. Based on this evidence, this is likely caused by vibrations of either the wind turbine generator’s (WTG) hydraulic system and or the transformer. The latter that is located inside the turbine on top of the foundation.

Furthermore, all correlation graphs display a certain level of noise that is likely caused by (i) slight errors in the temperature compensation; (ii) a causality of the combination of T1 and T2’s vertical tower strain on the foundation strain; (iii) the underlying fluctuating foundation vibration as illustrated in Figure 11, and (iv) the impact of yawing and the turbine’s control such as for example the individual pitch control algorithm.

Table 1 illustrates the normalised average dead load (compiled from the dead load condition identified in Figure 9 and Figure 10) of the tower and circumferential foundation strain sensor.

Results reveal a normalised average dead load value of 0.5 for T1, thus along the prevailing wind direction and oppositely an equal absolute maximum positive and negative strain was encountered in the observed period. This agrees with expectations as the deployed wind turbine has a predominant bi-directional wind-inflow condition. For T2, the mean is marginally shifted indicating a slight imbalance in the absolute maximum positive and negative strain. This is also expected due to an overall lower probability that the turbine is acting in either of the two directions. Different results are found for the circumferential foundation sensor. In fact, the mean is substantially shifted meaning that the sensor has observed higher tension stresses than compression stresses. This is in agreement with the design assumption; i.e., concrete has essentially no tensile strength, so the steel reinforcement takes up the strain, whereas during compression the concrete is able to withstand the stress, thus alleviating strain from the reinforcement [36].

For the shear link this analysis was not possible to execute, because in the limited time period the wind inflow was not opposing the predominant wind direction.

### 3.2. FE Comparison

It is overall challenging to verify the FE model as it is a stationary model, while the turbine is subjected to a stochastic wind inflow, hence characterised by a dynamic response. Further complexity arises from multiple other reasons, namely: (i) the turbine operates under different control regimes such as for example below and above rated, paired with an individual pitch control algorithm; (ii) there is currently no available access to supervisory control and data acquisition (SCADA) measurements, and (iii) the turbine’s nacelle orientation with respect to the sensor direction is unknown, although the power spectral density of the tower base FBG strain gauges can give a reasonable estimate. Given such challenges, the aim is to evaluate if the measured strain ranges agree with those expected from the FE model or alternatively by what magnitude the strain is under or overestimated.

**Figure 12:**
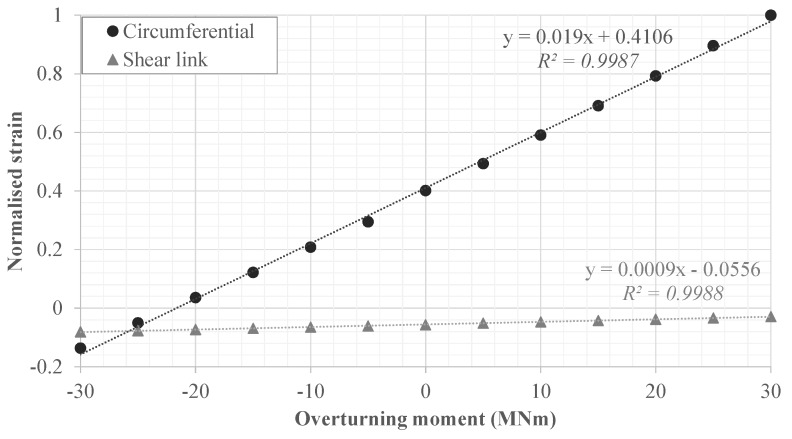
Normalised change in strain in shear link and circumferential rebars, extracted from the FE model. Positive overturning moments follow the wind vector given in Figure 1.

**Figure 13:**
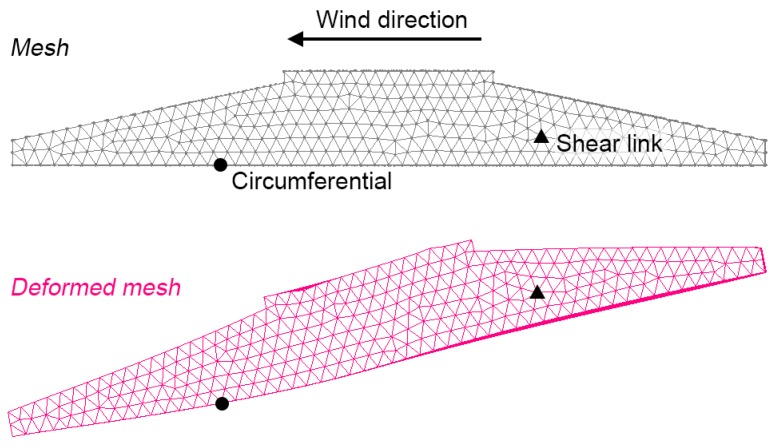
Mesh and sensor locations before and after loading (deformed mesh scaled by $10^{3}$).

Generally, the sensor data agrees with the simplified FE model, the results of which are shown in Figure 12 and Figure 13. Indeed, the circumferential strain sensor experiences a substantially higher strain magnitude than the shear link. The lack of deformation in the axial direction is highlighted by the deformed mesh in Figure 13 and this confirms operator’s existing knowledge. This is also the reason why the shear link strain in Figure 11 appears noisy in relation to the circumferential sensor’s measurements. The circumferential strain reaches 95 μϵ whereas the shear link reaches maximum strains of 6 μϵ. This is in agreement with the FEA’s data that suggest a reduction of the shear link strain by an order of magnitude in comparison to the circumferential rebar’s strain.

Figure 12 also illustrates that the foundation system favours positive (tensile) circumferential strains, regardless of the direction of the wind. The reason for this is the impact of the foundation, tower and ballast dead loads, and the fact that the soil below the foundation only supports compression (due to the lift-off boundary condition). The overturning moment zero crossing-point in Figure 12 cannot yet be directly compared with the measured data, as SCADA data (and hence knowledge of overturning moments) is not available at the time of writing.

## 4. Validation

**Figure 14:**
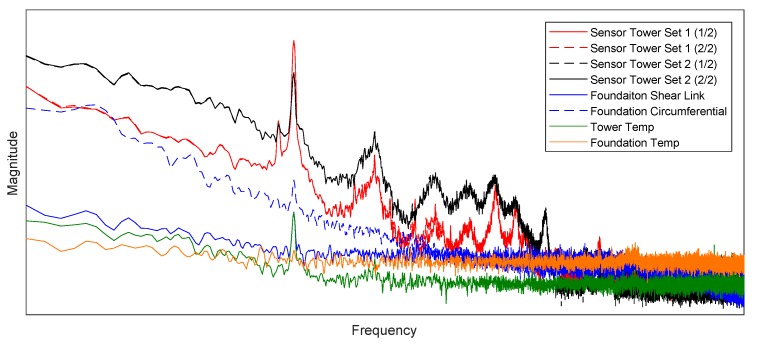
Power spectral density of strain and temperature sensors. Temperature sensor: raw data; FBG strain gauge: temperature compensated. Due to confidentiality, the *x* and *y* axes labels and ticks are removed.

Figure 14 illustrates the power spectral density (PSD) of the different strain and temperature signals allowing to identify structural modes, the rotor frequency (1P) as well as the rotor frequency’s harmonics (nP) [37,38]. Due to confidentiality agreements, the x and y axes data labels are not disclosed.

With regards to the installed tower FBG strain gauges, both tower pairs (strain gauges are 180 degrees apart) show an equal PSD. This is in agreement with Newtons’s third law of motion [39], verifying a correct installation with the sensors exhibiting identical strain transfers. The tower temperature sensor has a significantly different PSD, suggesting that the actual tower strain is not picked up in the signal; however, a dominant structural mode is picked up. In essence, it is challenging to completely isolate a temperature sensor from the structure’s vibration, but given that the power in the mode is significantly lower in magnitude compared to the power level observed in the strain sensors, this is acceptable. Further, the temperature sensors are filtered with a moving average within the window of 80 s, hence effectively eliminating these signals altogether. Temperature readings are therefore observed as fit for purpose for temperature compensation of the corresponding tower FBG strain gauges.

**Figure 15:**
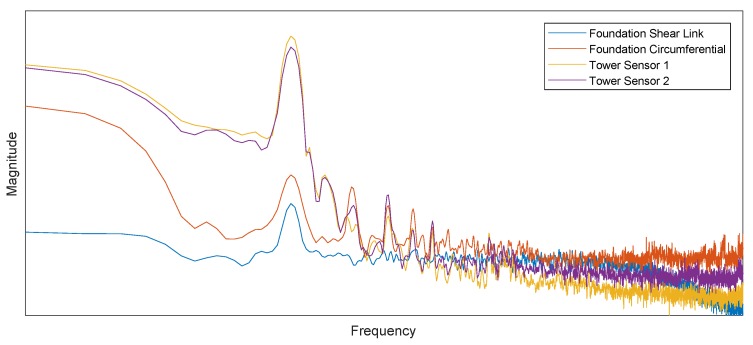
Power spectral density of foundation and bottom tower FBG strain gauges. Temperature sensor: raw data; strain gauge: temperature compensated. Due to confidentiality, the *x* and *y* axis labels and ticks are removed.

With regards to the foundation FBG strain gauges, both PSDs have an equal distribution, although the shear link has a lower overall magnitude. This difference agrees with the FE model as well as feedback from the foundation designer. Both foundation sensors further pick up the first tower mode, giving confidence in their readings. The foundation temperature sensor’s PSD picks up the first tower mode; however, similarly to the tower sensors on a much lower magnitude allowing confidence in its application for temperature compensation. In addition, at lower frequencies, the power in the foundation temperature signal (e.g., daily variations) is lower than the tower temperature sensor, which is expected as the temperature changes at a higher rate at the bottom tower section than in the buried foundation. Further, results are in agreement with findings of the PSD of the turbine’s emergency stop, executed as part of the commissioning as illustrated in Figure 15. This data illustrates well how the optical strain gauges detect the turbine’s propagating natural modes and their harmonics.

## 5. Discussion and Future Work

Although the sensor network was prepared for rough handling and substantial impacts during the installation and concrete pour, forces in the addressing fibre were much higher than expected. This resulted in the loss of 75% of the installed sensors. The knowledge gained during this installation will inform future work that aims to install optical sensor networks in civil engineering applications with similar complexity. Future work thus entails an investigation to determine forces along the addressing fibre and methods to guide cables in the foundation as well as options for interconnections. It is recommended that military optical connectors are considered for any future installations, although the issue in applying such connectors is their thick module diameter in comparison to the applied reinforcement bars. Alternatively, in order to maintain a slim connector thickness, the presented crimped interconnection may be trialled with additional material layers to enhance strength and resilience. One option may be to add an epoxy layer at the point of interconnection. The latter is likely to facilitate a slim connection while increasing overall strength; however, on the downside the installation time increases due to epoxy curing. Other options include to pre-cure concrete around the sensor and locations of cable interconnections.

With regards to the results and their validation, the object was to compare foundation sensor readings with their mirrored counterparts (placed 180 degrees apart in the foundation). This is a similar methodology to the validation of the tower strain gauges. However, because the mirrored strain gauges were lost, this straightforward validation could not be achieved.

Nevertheless, the sensor data agrees reasonably well with the simplified FEA’s results as demonstrated in Section 3, besides the ability to detect structural modes and harmonics as presented in Section 4. In order to gain a more detailed, accurate, and fully validated picture of the overall foundation loading, the project requires a successful repetition. Nevertheless, long-term data acquisition using the existing trial will be valuable to assess the internal health of the foundation, albeit limited to the locations covered by the surviving sensors. At the end of the turbine’s design life, sensor readings may be applied to aid repowering and lifetime extension decisions, given the difficulty to otherwise internally assess the wind turbine foundation’s structural integrity and cumulative fatigue damage. Here, local sensor data may be extrapolated in combination with the results generated by FEA.

## 6. Conclusions

This paper presents the results of a first-time demonstration of reinforcement strain monitoring within an onshore, concrete wind turbine foundation during operation. The sensor design, construction and field installation activities have been invaluable in the learning process to successfully design and deploy a structural health monitoring system for an onshore wind turbine foundation. The results presented may act as a valuable guide for similar projects. The results obtained have locally corroborated finite element models of the foundation and have shown a strong correlation with measured tower dynamics. The sensor data, even in its limited form, may prove valuable in lifetime extension or foundation redesign to safely reduce the fixed costs and environmental impacts of wind power.

## Figures and Tables

**Table 1:** Dead Load Condition.

Strain Sensor	Normalised Average Dead Load
Tower T1	0.5
Tower T2	0.48
Circumferential	0.38

**Figure A12:**
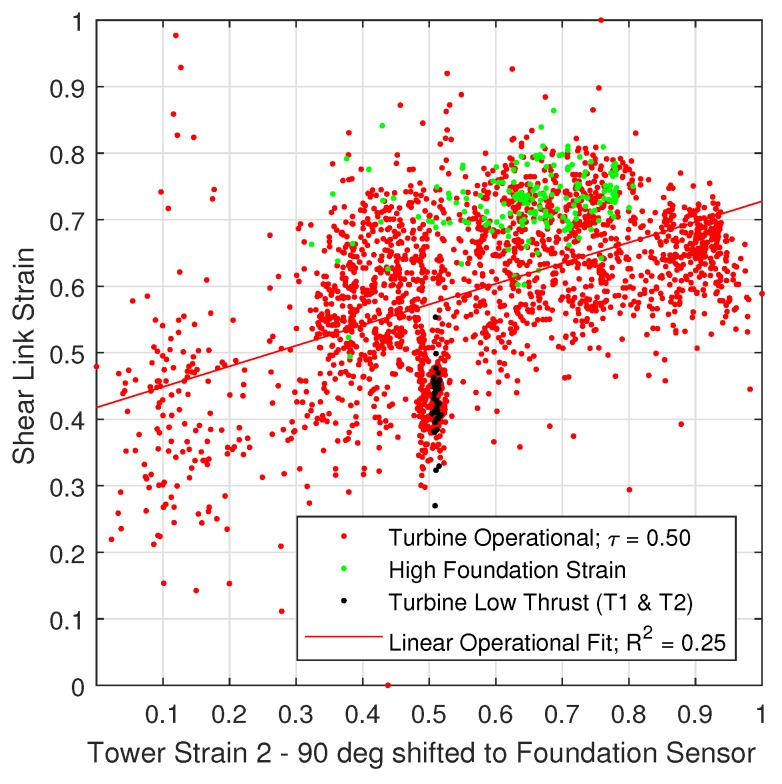
Normalised correlation of shear link strain with 90 deg rotated tower sensor.

## Abbreviations

The following abbreviations are used in this manuscript:

FE	Finite element
FBG	Fibre Bragg grating
FEA	Finite element analysis
ID	Inner diameter
OD	Outer diameter
CAD	Computer aided design
SCADA	Supervisory control and data acquisition
PSD	Power spectral density
UPS	Uninterruptible power supply
WTG	Wind turbine generator

## Appendix A

**Table A1:** Mechanical evaluation of sensor sensitivity.

Test Information	Test Method	Machine	Sample Stress/Strain Input	Sensor Sensitivity [nm/mϵ]
Metal packaged optical foundation strain gauge attached to metal sample (1/2)	Dynamic	Instron 8802	± 50 MPa/±0.25 mϵ at 0.25 Hz	0.82
Metal packaged optical foundation strain gauge attached to metal sample (2/2)	Dynamic	Instron 8802	± 100 MPa/±0.5 mϵ at 12 Hz	0.78
Tower FBG strain gauge attached to metal sample	Static	Testometric M350-10CT	4 × 30 MPa steps	0.89
